# Microporous Polymelamine Framework Functionalized with Re(I) Tricarbonyl Complexes for CO_2_ Absorption and Reduction

**DOI:** 10.3390/polym14245472

**Published:** 2022-12-14

**Authors:** Stefania Zappia, Elena Perju, Andrei Bejan, Adina Coroaba, Filippo Bossola, Juqin Zeng, Daniele Sassone, Luminita Marin, Silvia Destri, William Porzio

**Affiliations:** 1 Institute of Chemical Sciences and Technologies “G. Natta” Consiglio Nazionale delle Ricerche (SCITEC-CNR) via A. Corti 12, 20133 Milano, Italy; 2 “Petru Poni” Institute of Macromolecular Chemistry, Gr. Ghica Voda Alley, 41A, 700487 Iasi, Romania; 3 Institute of Chemical Sciences and Technologies “G. Natta” Consiglio Nazionale delle Ricerche (SCITEC-CNR) via C. Golgi 19, 20133 Milano, Italy; 4 Center for Sustainable Future Technologies CSFT@PoliTo, Istituto Italiano di Tecnologia, IIT Via Livorno, 10144 Torino, Italy

**Keywords:** porous organic polymers, melamine-based polymers, rhenium, CO_2_ absorption, CO_2_ electrochemical reduction

## Abstract

A mixture of polymeric complexes based on the reaction between Re(CO)_5_Cl and the porous polymeric network coming from the coupling of melamine and benzene-1,3,5-tricarboxaldehyde was obtained and characterized by FTIR, NMR, SEM, XPS, ICP, XRD, and cyclic voltammetry (CV). The formed rhenium-based porous hybrid material reveals a noticeable capability of CO_2_ absorption. The gas absorption amount measured at 295 K was close to 44 cm^3^/g at 1 atm. An interesting catalytic activity for CO_2_ reduction reaction (CO_2_RR) is observed, resulting in a turn over-number (TON) close to 6.3 under 80 min of test at −1.8 V vs. Ag/AgCl in a TBAPF_6_ 0.1 M ACN solution. A possible use as filler in membranes or columns can be envisaged.

## 1. Introduction

The dramatic climate change occurring during last decades is strictly connected with CO_2_ continuously increasing, and it has pushed researchers to prepare new materials suitable for the capture and sequestration of this gas, aside from the vegetable chlorophyll photosynthesis [1,2,3].

The European Community stated that almost 80% of the product’s environmental impact should be improved through material eco-design [4].

In this view, amorphous materials containing ultra-micropores have been recognized as a very promising candidate for CO_2_ capture due to their low density, high thermal and chemical stability, large surface area, tunable pore size, and structure [5].

Porous materials include a very broad range of different structures, from natural to synthetic, from inorganic to organic, and from crystalline to amorphous. Briefly, metal-organic frameworks (MOFs) represent a class of organic-inorganic hybrid solids with uniform framework structures built from organic linkers and inorganic metal nodes, or metal-containing clusters [6,7,8,9,10]. Otherwise, porous organic polymers (POPs) are organic macromolecules constructed exclusively from organic covalent bonds and are mainly composed of light elements (H, B, C, N, and O), hence they are usually lightweight materials. Due to the nature of the covalent bonds, POPs usually display very high stability compared with most MOFs, which are linked through coordination bonds [11,12,13,14,15]. And covalent triazine frameworks (CTFs) with both amorphous and crystalline structure [5,12,16,17].

By tuning the structural, electronic, and physical properties of the frameworks through direct synthesis and/or post-synthetic modifications, distinct catalytic properties as, for example, selectivity, can be modulated, making these materials versatile for the capture and reduction of CO_2_ gas. In particular, CO_2_ can be reduced with high efficiency to obtain valuable chemicals, such as CO, HCOOH, HCHO, CH_3_OH, or CH_4_ at cathode with highly efficient electrocatalysts for the sustainable energy conversion/utilization and the carbon recycle [18]. Moreover, recent studies have pointed toward pyridinic nitrogen as an interesting active site for CO_2_ reduction [19] and evidence that the metal centers are in fact crucial for the CO_2_ to CO transformation [20].

Specifically, CTFs obtained by the polymerization of aromatic nitrogen-rich compounds have been investigated as porous materials with promising application form gas adsorption and/or separation to heterogeneous organic electro- and photocatalysis, exploiting their large surface area and their thermal, chemical, and mechanical stability [21,22,23,24,25]. In this framework, melamine-based POPs, synthetized through the one-pot catalyst-free Schiff base reaction between melamine and 1,3,5-tricarboxaldehyde and related derivatives, have been studied as nitrogen-rich microporous materials for the development of CO_2_-capture adsorbers [25,26].

In this respect, the use of rhenium (Re) derivatives is among the most robust and selective catalysts for the electrochemical reduction of CO_2_ to CO, both as mononuclear isolated complex [27,28,29,30,31] as well as organic-inorganic hybrid polymer-based materials in heterogenous conditions mostly based on biphenylene moiety as ligand [14,32,33].

Since, to the best of our knowledge, no examples combining melamine-based porous materials with rhenium complexes, to obtain hybrid organic-inorganic systems capable of absorbing and reducing CO_2_ to valuable chemicals, have been reported, with the present paper we like to reduce this gap, thus synthetizing a new material in which a molecular electrocatalyst, based on the Re(I) tricarbonyl unit, was immobilized on POPs. Melamine is a natural, very common, and cheap product and melamine-based POPs are efficient CO_2_ absorbers, hence this new compound could offer the possibility to capture and reduce CO_2_ through a single material.

A melamine-based porous polymeric network has been synthetized by using the one-pot catalyst-free Schiff base chemistry, between melamine and benzene-1,3,5-tricarboxaldehyde. Then, the post-metallation reaction of the POPs with Re(CO)_5_Cl, as organometallic precursor, was realized, exploiting the presence of nitrogen atoms as ligands for the transition metal. The resulting Re@POPs were extensively characterized by means of Fourier-transform infrared spectroscopy (FTIR), X-ray photoelectron spectroscopy (XPS), X-ray powder diffraction (XRD), inductively coupled plasma optical emission spectroscopy (ICP-OES) analysis, and cyclic voltammetry (CV), and then they were tested for gas absorption and electrocatalytic reduction of carbon dioxide.

The obtained Re@POP exhibits CO_2_ absorption properties, even though reduced with respect to the precursor POP synthetized in our laboratory. Additionally, it shows interesting catalytic activity for (CO_2_RR) resulting in a low turn over-number (TON).

## 2. Materials and Methods

### 2.1. Material Preparation

Melamine, benzene-1,3,5-tricarbaldehyde, and Re(CO)_5_Cl were obtained from Sigma-Aldrich and used as received, without further purifications. All manipulations of air and moisture sensitive materials were conducted under a nitrogen atmosphere on a dual manifold Schlenk line. The glassware was oven-dried prior to use. DMSO (99.5% Sigma Aldrich, St. Louis, MO, USA) was degassed while toluene (99.5% Sigma-Aldrich) was distilled on Na(s) and CH_2_Cl_2_ on P_2_O_5_, both under a N_2_ atmosphere.

#### 2.1.1. Porous Organic Polymers (POPs) Synthesis (Coupling of Benzene-1,3,5-Tricarboxaldehyde with Melamine)

A dried Schlenk flask was charged with melamine (85 mg, 0.6784 mmol), benzene-1,3,5-tricarbaldehyde (110 mg, 0.6784 mmol) and dimethyl sulfoxide (4.5 mL). After degassing by three freeze-pump-thaw cycles, the reaction mixture was heated at 180 °C for 24 h under N_2_ atmosphere. Then 2 mL of DMSO were added and the reaction was heated at 180 °C for another 48 h (**POP1**). In another run (**POP2**) 6.5 mL of DMSO were added at the beginning before the degassing process. In both cases, the residue was washed with excess of acetone, dichloromethane and THF, followed by Soxhlet extraction with THF to remove the decomposition products of DMSO and the eventually non-reacted reagents, not bound to the network. Further, the product was dried in an oven at 73 °C for 4 days, leading to an off-white powder in a yield varying from 71 (**POP1**) to 63% (**POP2**).

#### 2.1.2. Typical Synthesis of Re@POP

Solid POP was heated under vacuum at 130 °C for 5 h to remove all the side-products coming from DMSO degradation. After that, 44.6 mg was transferred to Schlenk tube equipped with a magnetic stir bar and a reflux condenser. Re(CO)_5_Cl (46.6 mg, 0.129 mmol) was added and the reaction mixture was suspended in toluene (10 mL) and heated at reflux temperature for 3 h, when a slight color change was observed from off-white to pale yellow. The resulting powder was separated from the reaction solution, washed with freshly distilled toluene (3 × 5 mL), dichloromethane (3 × 5 mL), then dried under vacuum and stored under nitrogen atmosphere at room temperature.

### 2.2. Material Characterizations

#### 2.2.1. Attenuated Total Reflectance (ATR)-Fourier-Transform Infrared Spectroscopy (FTIR)

FTIR spectra were recorded on a FT-IR Bruker Vertex 70 Spectrophotometer (Bruker, Billerica, MA, USA) and on a PerkinElmer Frontier instrument (PerkinElmer, Waltham, MA, USA) equipped with an ATR accessory with a diamond/ZnSe crystal. The IR spectra were acquired between 4000 and 400 cm^−1^.

#### 2.2.2. ^13^C Cross-Polarization Magic-Angle-Spinning (CPMAS) NMR

The NMR spectra were recorded with an FT-NMR AvanceTM 500 (Bruker BioSpin S.r.l., Billerica, MA, USA) with a superconducting ultrashield magnet of 11.7 Tesla operating at 125.76 MHz ^13^C frequency. The following conditions were applied for the cross-polarization (cp.av) experiments: repetition time (relaxation delay) = 2 s, ^1^H 90 pulse length = 4.2 μs, contact time = 1 ms, and spin rate at magic angle (MAS) = 12 kHz. The compounds were placed in a zirconium rotor: 80 μL volume, 4 mm in diameter and 18 mm high. The chemical shifts were recorded relative to glycine standard, previously acquired (C=O signal: 176.03 ppm, relative to tetramethyl silane reference).

#### 2.2.3. Scanning Electron Microscopy (SEM)

The analysis was performed with a field emission Scanning Electron Microscope SEM EDAX–Quanta 200, operated at an accelerating voltage of typically 20 keV.

#### 2.2.4. Electrochemistry: Cyclic Voltammetry (CV)

The as-prepared catalysts were coated onto a carbon paper (GDL; SIGRACET 28BC, SGL Technologies, Lagos, Nigeria) in order to enable the electrochemical evaluation of the powder-like materials toward the CO_2_RR. 4.5 mg of catalyst were dispersed in 160 µL of isopropanol with the addition of 20% *w*/*w* of carbon black nanoparticles and 10% in *w*/*w* of Nafion as binder by means of sonication for 20 min. The obtained slurry was then drop-casted on the GDL and dried in air overnight. Each electrode has a catalyst loading of 1 mg∙cm^−2^. The electrochemical characterization was firstly performed through cyclic voltammetry (CV) in a single-chamber cell at room temperature with a Metrohm Multi Autolab/M101 potentiostat. The working electrode was a catalyst-coated carbon paper with a geometric area of 0.15 cm^2^. A Pt wire was used as the counter electrode and an Ag/AgCl (3 M NaCl) was used as the reference. The measurements were performed from 0.0 to −2.0 V vs. Ag/AgCl at a scan rate of 10 mV∙s^−1^ firstly in N_2_-purged and then in CO_2_-saturated 0.1 M TBAPF_6_ acetonitrile solution. The gas flow rate was 5 mL∙min^−1^.

#### 2.2.5. Chronoamperometry (CA)

Tests were performed with a CHI 760D (CH Instruments, Inc, Austin, TX, USA) potentiostat in a H-type cell in which two compartments were separated by a cation exchange membrane (Nafion™ Membrane N117, Sigma-Aldrich, St. Louis, MO, USA) employing a 0.1 M TBAPF_6_ acetonitrile solution at the cathodic side and a 0.1 M KHCO_3_ water solution at the anodic side. A catalyst-coated carbon paper of 4.5 cm^2^, with the same composition of that for the CV, was used as the working electrode, a Pt foil as the counter and an Ag/AgCl (3M NaCl) as the reference. Gas-phase products were analyzed on-line by a micro gas chromatograph (µGC, Fusion^®^, INFICON, Bad Ragaz, Switzerland) with two channels containing a 10 m Rt-Molsieve 5A column and an 8 m Rt-Q-Bond column, respectively. Both channels are equipped with a microthermal conductivity detector (micro-TCD). The inlet of the µGC equipment was connected to the cathodic side of the electrochemical cell through a GENIE filter to remove the humidity from the gas. During the CA measurements, a constant CO_2_ flow rate of 15 mL∙min^−1^ was maintained inside the solution to carry out the product to the µGC. The total µmol of the CO_2_RR products were quantified and used to calculate the values of TON (TON = total µmol of CO/ total µmol of Re quantified by ICP).

#### 2.2.6. Inductively Coupled Plasma Optical Emission Spectroscopy (ICP-OES)

Samples were entirely weighed (between 4 and 10 mg of powder) and transferred into a Teflon vessel. Ultrapure sulfuric acid (5 mL) was added to each vessel, which was closed by using a dynamometric key, and all the samples were submitted to mineralization assisted with microwaves. The vessels were cooled to room temperature, opened, and filled with ultrapure nitric acid (5 mL), then mineralized again by using microwaves. The obtained solutions were diluted to 50 mL with distilled water and analyzed by ICP-OES by using a Perkin Elmer Optima 8300 instrument (Waltham, MA, USA). A calibration curve was obtained by preparing different certified standard concentrations of Re (10, 20, 40 mg∙L^−1^), in order to quantify the Re amount in the samples.

#### 2.2.7. X-ray Photoelectron Spectroscopy (XPS) Analysis

X-ray photoelectron spectra were collected on a Kratos Analytical Axis NOVA instrument (Nova Instrument, Rehovot, Israel) using monochromatic Al Kα X-rays source (hν = 1486 eV), 20 mA current and 15 kV voltage (300 W), and base pressure of 10^−8^ to 10^−9^ Torr in the sample chamber. The incident monochromated X-ray beam was focused on a 0.7 × 0.3 mm^2^ area of the surface. The XPS survey spectra were collected in the range of 5 ÷ 1200 eV with a resolution of 1 eV and a pass energy of 160 eV. The high-resolution spectra for all the elements identified from the survey spectra were collected using a pass energy of 20 eV and a step size of 0.1 eV. The binding energy value was calibrated by the C1s peak (285 eV). The recorded spectra were always fitted using Gauss-Lorentz curves to determine more accurately the binding energy of the different element core levels. The curve deconvolution of the obtained XPS spectra was analyzed using the ESCApe software.

#### 2.2.8. X-ray Powder Diffraction (XRD) Analysis

Concerning the collection of the wide-angle X-ray diffraction (WAXS) pattern, the material powders were finely grinded in glove-box, to minimize the preferred orientation effects, pressed in sample-holder and mounted into an Anton-Parr Camera part of a Siemens D-500 diffractometer equipped with energy detector (Vortex of SII Nanotechnology, Northridge, CA, USA) and Soller slits of 2°. The measurements were performed at 293 K in Bragg-Brentano geometry. The wavelength was Cu-Kα radiation (λ = 0.154 nm), the operating voltage and current were 40 kV and 40 mA, respectively. Data were collected from 5° to 45° (2θ) in steps of 0.05° (2θ) for 6 s.

#### 2.2.9. Gas Absorption Analysis

The analyses were performed on a Micromeritics ASAP 2020 physisorption instrument at 295 K until an absolute pressure of about 786 mmHg. The samples were degassed at 110 °C for 3 h before each analysis.

## 3. Results

### 3.1. Synthesis

**Scheme 1:**
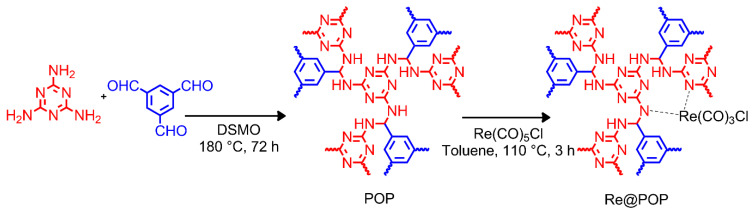
Synthetic route to the melamine-based polymer network (porous organic polymer, POP) through catalyst-free one-pot Schiff base type reaction, and subsequent Rhenium-based derivative of POP (Re@POP) formation.

The targeted porous organic polymers (POPs) were synthesized using modified procedures [25,26] based on the condensation of melamine and benzene-1,3,5-tricarboxaldehyde under the solvothermal conditions, using dimethyl sulfoxide (DMSO) as the reaction medium (Scheme 1).

Previous investigations proved that DMSO decomposes at high temperature forming formaldehyde. The possibility of its reaction with the free amino groups of melamine to form methylol groups and further an ether linkage and a methylene linkage via condensation reaction, was considered too [34,35,36].

**Figure 1:**
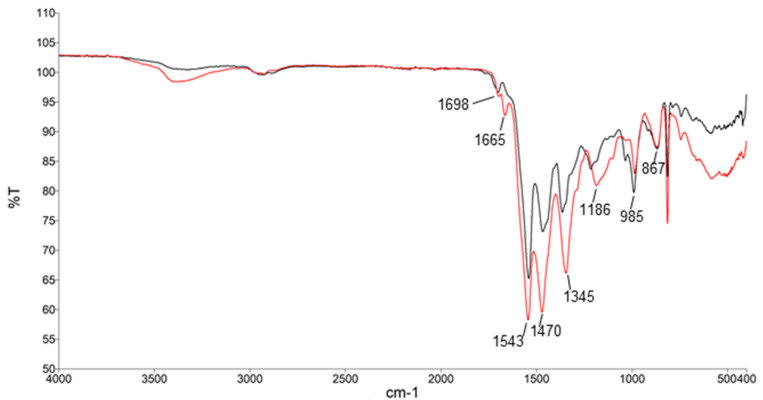
FTIR spectra of **POP1**(black line) and **POP2** (red Line).

The structure of the products has been confirmed by FTIR and solid-state NMR spectra. As can be seen in Figure 1 and Figure S1, the sharp characteristic absorption bands of the primary amine groups of melamine from 3420 and 3470 cm^−1^ (NH_2_ stretching) and 1650 cm^−1^ (NH_2_ deformation)), and aldehyde groups of benzene-1,3,5-tricarboxaldehyde (2870 cm^−1^ (C-H stretching of CHO) and 1690 cm^−1^ (C–O stretching)) were absent or drastically reduced after the polymerization. A weak band at 1698 cm^−1^ in **POP1** indicates some carbonyl group left, while in **POP2** a feeble band at 1665 cm^−1^(N-H deformation) shows the presence of amine groups. These features emphasize the successful reaction between the amine and aldehyde groups. The distinct bands corresponding the quadrant (1550 cm^−1^) and semicircle stretching (1480 cm^−1^) of the triazine ring were present in both spectra of the melamine monomer and the POP polymer, indicating the incorporation of the melamine into the network. The absence (**POP1**) or a dramatic decreasing (**POP2**) of the characteristic sharp band of imine linkage around 1600 cm^−1^ (Ar-C=N- stretching) indicates that the hyper-crosslinking was mainly controlled by the predominant formation of the aminal bonds (NH-C-NH) which C–N stretching band from secondary N-H is at 1185 cm^−1^, as other studies assumed [26,36,37,38,39].

**Figure 2:**
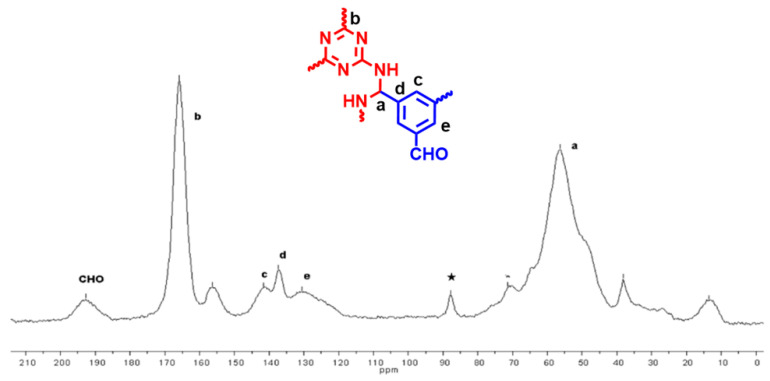
Solid-state ^13^C CP/MAS NMR spectrum of POP, asterisks indicate peaks arising from spinning side bands.

Solid-state ^13^C CP/MAS NMR (Figure 2) displays a strong resonance at 166 ppm attributed to the aromatic carbon of triazine ring, and the signals at 130, 137, and 141 ppm, which can be correlated to the aromatic carbons of the benzene ring. The resonance at 56 ppm was attributed to the carbon atoms of the aminal units resulted from reaction between amino and aldehyde groups. A weak carbonyl peak could be also observed at 193 ppm, attributed to unreacted aldehyde groups, consistent with FTIR analysis. No imine type carbon resonance at 160 ppm is observed, indicating the absence of imine bond in the polymer network [23]. Besides, the signal at 38 ppm corresponds to the residual DMSO in the polymer while the signal at 70 ppm is due to methylene groups in ether linkage and the shoulder at 53 ppm overlapping the signal of carbon of and it can be attributed to methylene linkages. Both derive from the reaction of amine and formaldehyde, coming from DMSO degradation [36]. At the highest fields near 14 ppm, the signal attributable to methyl carbon of alkane (methane absorbed in the hyper-crosslinked polymer) could be observed in **POP2** sample. This side-product too originates from the DMSO degradation due to the reaction with hydroxyl radicals [36]. These features allow us to strength the idea that the polymer network is based mainly on aminal connections instead imine bonds, in agreement with the active amino groups of the electron-rich triazine ring.

**Figure 3:**
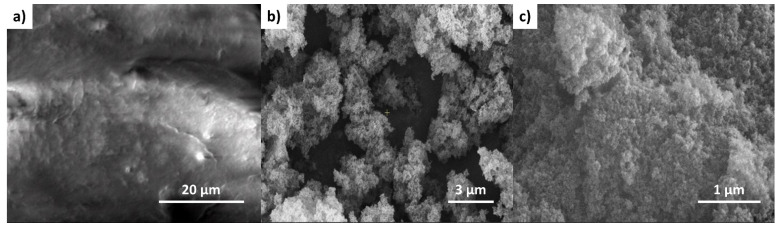
SEM images of **POP2** taken at different magnification, (**a**) 20 µm, (**b**) 3 µm, and (**c**) 1 µm.

SEM images on POP sample are shown in Figure 3, taken at increasing magnification in different areas indicated micro-pores, in agreement with the absorption analysis (see below). The different materials from **POP1** and **POP2** display almost comparable porosity.

Due to the presence of nitrogen atoms in the aminal units of the POP structures, the post-synthetic modification of this framework, in particular the post-metalation method was proposed to covalently bind “Re(CO)_3_” units, thus resulting in a heterogeneous material for CO_2_ reduction. Treatment of POP with Re(CO)_5_Cl in toluene solution under reflux for 3 h gave rise to a pale-yellow powder Re@POP, (Scheme 1) which was purified with solvent exchange to remove the starting material. ICP-OES analyses, performed onto both runs, indicated Re amount of 21.9 ± 1 % and 23.2 ± 1 % for **POP1** and **POP2** samples, respectively. These values compared with C, H, and N content, checked by elemental analysis (Scheme 1), are compatible with a Re atom per POP unit within the uncertainties due to the chemical array non-univocally defined (see Supplementary Materials). Moreover, the presence of “Re(CO)_3_(N^N)” (where N^N means only a coordination) units was indicated by FTIR characterization and confirmed by XPS analysis).

**Figure 4:**
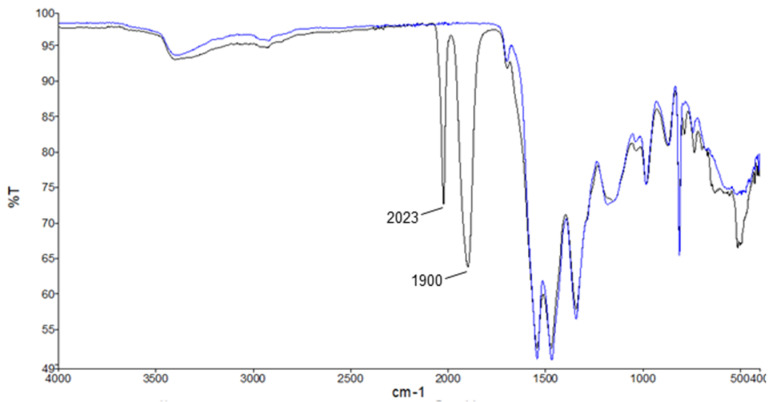
FTIR spectra of POP sample (blue line) and Re@POP (black line).

In particular, FTIR spectra (Figure 4) display two new ν(CO) bands at 2023 and 1900 cm^−1^, corresponding to the CO vibrational stretching of the carbonyl ligands on the mononuclear rhenium complexes. It is interesting to note that these bands are different to those observed for the starting material, Re(CO)_5_Cl (2037 and 1959 cm^−1^), and POP in the same conditions (Figure 1). This features (red shift of C=O stretching bonds and broadening of quadrant stretching at 1550 cm^−1^) indicate that the coordination at nitrogen atoms of the polymer framework and, thus the formation of mononuclear Rhenium derivatives based on “Re(CO)_3_” moieties, has actually occurred.

The solid-state ^13^C CP/MAS NMR spectra are shown in Supplementary Materials (Figure S2).

### 3.2. X-ray Photoelectron Spectroscopy (XPS) Analysis

**Figure 5:**
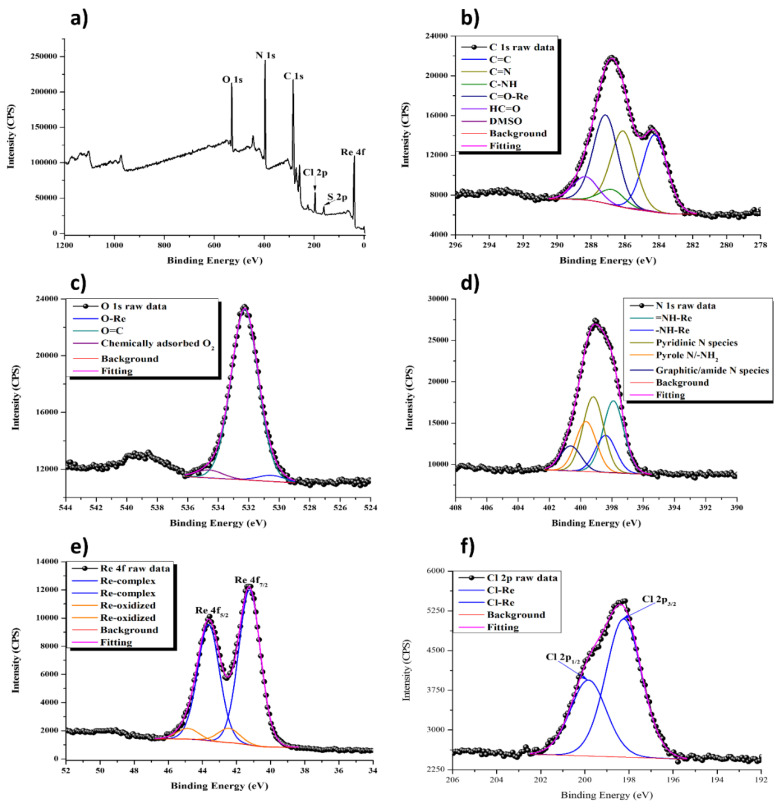
XPS spectra of the **Re@POP1** sample, (**a**) wide scan; (**b**) C 1s high resolution spectrum; (**c**) O 1s high resolution spectrum; (**d**) N 1s high resolution spectrum; (**e**) Re 4f high resolution spectrum; (**f**) Cl 2p high resolution spectrum.

As for the rhenium-based POP reported in ref. [14], the functionalization of the covalent organic polymers with chelating nitrogen ligands led to the isolation of Re-based POP. To shed further light on the nature of Re(POP), an in-depth XPS analysis was carried out (Figure 5).

The wide-scan spectrum of Re-based **POP1** (hereafter **Re@POP1**), reported in Figure 5a, showed the presence of the O 1s, N 1s, C 1s, Cl 2p, Re 4f, and S 2p absorption bands. While O, N, C, Cl, and Re can be correlated to the structure of the porous Re-based hybrid material, the presence of S highlights the presence of traces of DMSO on the surface of the framework, which were identified at bulk level by the solid-state ^13^C CP/MAS NMR measurements too.

The deconvolution of the high-resolution spectrum of the C 1s band, see Figure 5b, showed six distinct contributions at 284.2 eV, 285 eV, 286.1 eV, 286.7 eV, 287.1 eV, and 288.3 eV, respectively. The signals at 284.2 eV and 288.3 eV were attributed to aromatic C and terminal unreacted HC=O groups originally within the structure of the benzene-1,3,5-tricarboxaldehyde. The band at 286.1 eV was correlated to the aromatic C double bonded with N originally in the structure of melamine while the signal at 286.7 eV was assigned to C in C-N bonds that are formed over the course of the synthesis of the POP polymer. The band at 287.1 eV was attributed to the carbonyl ligands bonded to the Re metallic core. A quite weak band that appears at 285 eV was correlated to the before mentioned trace of DMSO [40,41].

The O 1s high resolution spectrum of the **Re@POP1** sample (Figure 5c) contained contributions from three components which appeared at 530.5 eV, 532.3 eV and 534.6 eV. The main deconvolution peak within the structure of the O 1s signal was correlated to O within C=O bonds which can be either from unreacted aldehyde moieties from benzene-1,3,5-tricarboxaldehyde or from the carbonyl ligands linked to the Re metallic core. The weak contribution of O in S=O groups from DMSO appears at values very close to the main deconvolution peak [40,41] and as such could not clearly be separated. The contribution at the lowest binding energy is in the range characteristic for metal oxides indicating that at the surface of **Re@POP1** quite small amount of ReO_2_ has been formed. This behavior has been encountered in previous XPS studies of rhenium-based hybrid materials and has been attributed to oxidation of the metal that occurs at the topmost surface layer. While during the synthesis of **Re@POP1** was dried under vacuum and stored in an inert atmosphere the compound has come into contact with trace amounts of O_2,_ which have oxidized Re atoms on the surface to ReO_2_. This phenomenon was only detected by XPS both due to the fact that the other analysis techniques involved bulk measurements and due to the technique capabilities to probe the very topmost surface layer of the analyzed samples. The contribution at the highest binding energy appears at values, which reference literature have indicated that it is characteristic for chemically adsorbed O_2_ [41,42,43,44]. This is thus further proof of **Re@POP1** coming into contact with trace amounts of O_2_ and to the fact that the framework is able to absorb gasses on its surface.

With regard to the N 1s signal (Figure 5d) the analysis was able to reveal that five contributions at 397.9 eV, 398.4 eV, 399.2 eV, 399.7 eV, and 400.6 eV, are present. The two contributions situated at the lowest binding energies present values consistent with amines and aromatic N coordinated with metals [45] and as such were attributed to the nitrogen in -NH-Re (Rhenium coordinated to the aminal nitrogen) and =N-Re moieties (Re coordinated to melamine ring). The three contributions that appear at the highest binding energies were assigned to pyridinic N species, pyrrole N or –NH_2_, and graphitic/amide N species that are formed as a result of the polymerization of POP [46].

The Re 4f high resolution spectrum of the **Re@POP1** sample (Figure 5e) presented the characteristic splitting of 4f5/2 and 4f7/2 and the shape of the peak included four distinct contributions allowing for the identification of two distinct compounds. At the lower binding energy of 41.2 eV the signal of the coordination complex of Re is located, which is consistent with data presented in literature for similar compounds [32,47]. At the higher binding energy, a contribution consistent with Re in a high oxidation state bonded to a high electron withdrawing element indicates the presence of ReO_2_ [48,49,50,51]. These results confirm the information obtained from the XPS analysis of O, as well as the fact that the oxidation of the central metallic core of Re has occurred.

The interpretation of the Cl 2p signal of **Re@POP1** sample (Figure 5f) is straightforward as the doubled splitting of 2p1/2 and 2p3/2 showed the presence of only two contributions at 198.2 eV and 199.8 eV indicating that only one type of Cl containing compound is present. This can of course be assigned to the Cl- ligand in the metallic complex of **Re@POP1** as indicated by reference literature of similar compounds [32,33].

The XPS analysis of Re-based **POP2** sample (hereafter **Re@POP2**) displays the same features of **Re@POP1**, hence the previous discussion applies. The related figures are reported in the supplementary materials (Figure S2). Briefly, the wide scan spectrum of **Re@POP2** (Figure S2a) revealed the presence of same elements (O 1s, N 1s, C 1s, Cl 2p, Re 4f, and S 2p). Apart from the structure unique peaks, the deconvolution of the C 1s high resolution spectrum showed two additional peaks although in traces, which were ascribed to adsorbed CO_2_ and dichloromethane (Figure S2b). In the case of the **Re@POP2** sample, the oxygen signal (Figure S2c) is broader due to a higher degree of oxidation that occurs in the sample. Entrapped NO_x_ species again in traces may also be visible in the oxygen and nitrogen signals (Figure S2d) due to the appearance of a new peak as compared to the **Re@POP1** sample. The Re oxidation here is more pronounced as it can be seen from the deconvolution of the Re 4f signal (Figure S2e) possibly due to different exposure of N type coordination centers (e.g., amino, imino, and aminal). The deconvolution of the Cl 2p high resolution spectrum (Figure S2f) indicates the existence of some dichloromethane residue entrapped in the molecular structure from the synthesis.

### 3.3. X-ray Powder Diffraction (XRD) Analysis

**Figure 6:**
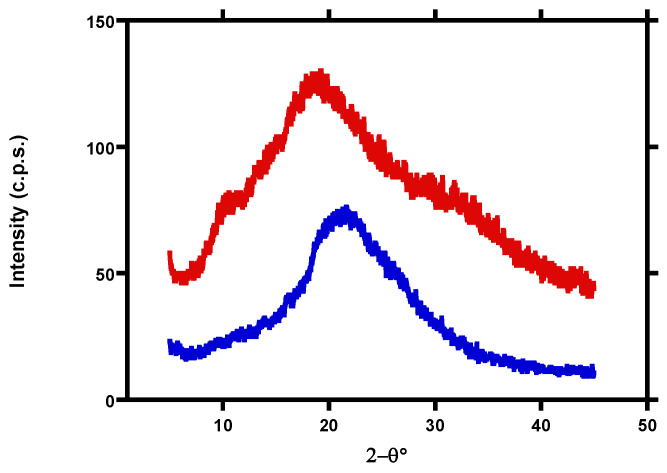
Powders XRD of **POP** (blue line) and **Re@POP** (red line).

In Figure 6 the XRD spectra of **POP1** and **Re@POP1** powders are reported. As largely expected in view of the undefined molecular assembly, POP profile is largely amorphous as shown by the broad peak centered at 21.20° (2θ), corresponding to d = 0.42 nm [35]. Additionally, Re-derivative shows large amorphous character, but the main bump is significantly shifted towards lower angles (18.5°), which d-spacing is close to 0.48 nm; a small bump is also detected near to 10.3°, which d-spacing is 0.86 nm. These remarks indicate that Re presence enlarge the overall arrangement, allowing also possible Re-Re contacts near to 0.9 nm. All these observations apply to both runs.

**Figure 7:**
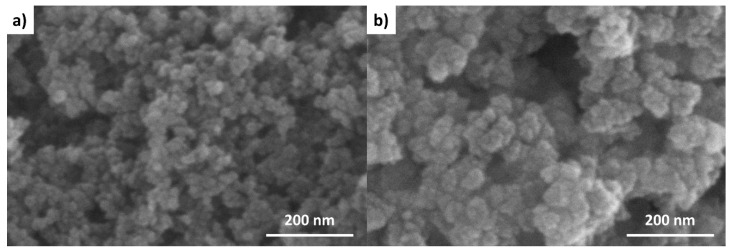
SEM images of (**a**) **Re@POP1** and (**b**) **Re@POP2**, differently obtained POP.

Further SEM analysis was performed, while at medium magnification, 2µ, differences between **POP** and **Re@POP** remain feeble (Figure S9), as shown in supplementary materials, high magnification reveals some differences showing the ultimate morphological details; in Figure 7 both samples are shown, they are characterized by porous aspect with significantly different globular features, namely **Re@POP1** display smaller aggregates compared to **Re@POP2**, typically from 10–20 nm up to 30–50 nm, respectively. This observation can be promptly related to different absorption properties (see below).

### 3.4. Electrochemical Studies

**Figure 8:**
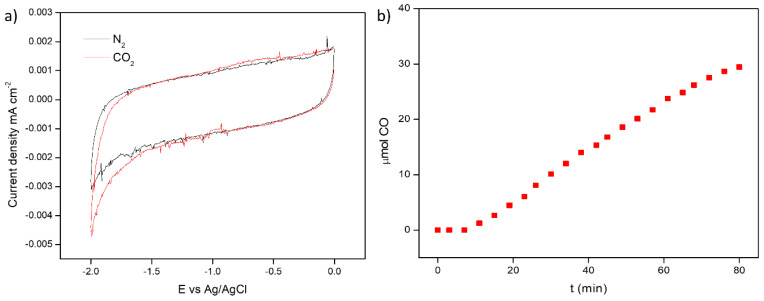
(**a**) Cyclic voltammetry tests on the electrode in N_2_-purged and CO_2_-saturated 0.1 M TBAPF_6_ acetonitrile solutions; (**b**) CO quantification during a chronoamperometric test in an H-cell at −1.8 V vs. Ag/AgCl in CO_2_-saturated 0.1 M TBAPF_6_ acetonitrile solution.

From the cyclic voltammetry test performed in aprotic solvent, 0.1 M TBAPF_6_ acetonitrile solution, the affinity of the catalyst for CO_2_RR is investigated purging into the solution firstly N_2_ and then CO_2_ while monitoring the current during the potential scan. As seen in Figure 8a, as switching from N_2_ to CO_2_ atmosphere, a significant increase in current density is registered from −1.0 V vs. Ag/AgCl and it becomes more notable at more negative potentials. Due to the nature of the aprotic solvent and the absence of external proton source, the current increase can be correlated directly to the activity towards the CO_2_RR happening at the catalyst/electrolyte interface. In order to quantify the catalytic activity, a chronoamperometric test was performed at −1.8 V vs. Ag/AgCl for 80 min, as shown in Figure 8b. During the experiment, the CO quantity increases as the function of time, indicating the continuous CO_2_RR to CO and confirming the presence of the active Rhenium-based derivatization of the POP. At the end of test, a total amount of 29.44 µmol of CO was obtained, resulting in a TON of 6.3, evaluated according to the specifics detailed in experimental section.

### 3.5. Gas Absorption Analysis

**Figure 9:**
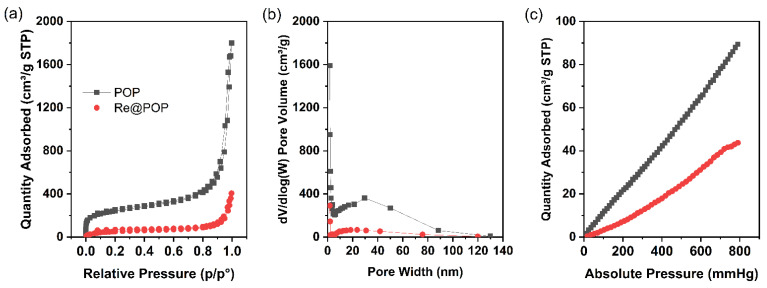
(**a**) N_2_ physisorption isotherm and (**b**) BJH pore volume and collected at 77 K on POP and Re@POP. (**c**) CO_2_ physisorption isotherms collected at 295 K on POP and Re@POP.

According to the IUPAC classification, Refs. [52,53] both the N_2_ physisorption isotherms of the bare and the Re@POP materials are tentatively of the type I (Figure 9a), which is characteristic of essentially microporous materials with relatively limited external surface area. Indeed, micropores make roughly the 25% of the total area of bare POP and no hysteresis loops can be seen in both isotherms. In Table 1 are summarized the main morphological data of both samples. Although maintaining the same shape of the isotherm, Re@POP has a markedly lower specific surface area compared to the bare POP (183 m^2^/g vs. 813 m^2^/g, respectively), accompanied by a drastic reduction in the pore volume (Figure 9b). Since the decrease in micropores area alone is not enough to justify such drop, this is consistent with the deposition of Re on the external surface of Re@POP with no variation of the overall morphology of material, in agreement with XPS experiments. It is worth noting that the behavior of both the POP runs is quite similar and the same applies to **Re@POP1** and **Re@POP2**.

The CO_2_ adsorption capacity was measured at 295 K until about atmospheric pressure. From the isotherms shown in Figure 9c, it is evident that the presence of Re inhibits the CO_2_ adsorption capacity which halves from 89.5 cm^3^/g to 43.7 cm^3^/g (Table 1). Both the values are in line with those found in the literature for similar materials [43,54]. To note, in both cases there is a linear relationship between the CO_2_ adsorption capacity and the pressure, meaning that only physical adsorption occurs [55].

## 4. Conclusions

A Rhenium-functionalized polymer, originating from the reaction between Re(CO)_5_Cl and the porous organic polymer (POP) obtained by the coupling between melamine and benzene-1,3,5-tricarboxaldehyde, has been prepared and fully characterized by FTIR, CPMAS NMR, ICP, SEM, XPS, XRD, cyclic voltammetry, and gas-absorption. Slight modifications of the condensation conditions yield porous polymeric network with different porosity and exposure to metalation of N-groups suitable to coordinate. In fact, a detailed XPS analysis confirmed that the microporous material is constituted by monomeric Re derivative variously coordinated to amine, imine (belonging to melamine ring), and aminal groups. The porous materials display significant CO_2_ absorption and capability of reduction to CO, particularly in non-aqueous medium. Monomeric Re derivative shows interesting catalytic activity for (CO_2_RR) too, resulting in a turn over-number (TON) close to 6.3 under 80 min of test at −1.8 V vs. Ag/AgCl in a TBAPF_6_ 0.1 M ACN solution.

The possibility of preparing by simple synthesis of microporous polymeric networks suitable for both CO_2_ absorption and reduction to CO has been reported envisaging the use of column filling, possibly overcoming the time-stability by substituting Re with alternative transition metals such as Co, Ru, and Cu.

## Figures and Tables

**Table 1:** N_2_ and CO_2_ physisorption results on POP and Re@POP *.

Entry	Specific Surface Area (m^2^/g) ^a^	Micropore Area (m^2^/g) ^a,b^	Pore Volume (cm^3^/g) ^a^	Pore width (nm) ^a^	CO_2_ Adsorbed (cm^3^/g) ^c^
POP	813	228	2.70	16.8	89.5
Re@POP	183	10	0.57	22.8	43.7

* Reported values correspond to sample Re@POP1 which properties are comparable with Re@POP2. ^a^ calculated from N_2_ physisorption at 77 K; ^b^ calculated by the t-plot method; ^c^ values of CO_2_ adsorbed at 790 mmHg and 295 K.

## Data Availability

Not applicable.

## Supplementary Materials

The following supporting information can be downloaded at: https://www.mdpi.com/article/10.3390/polym14245472/s1, Figure S1: FTIR spectra of benzene-1,3,5-tricarboxaldehyde, melamine, and COP. Figure S2: Solid state 13C CP-MAS NMR spectrum for COP and Re@COP. ICP-OES and Re content calculation. Figure S3: Wide scan XPS spectrum of the Re@COP2 sample. Figure S4: C 1s high resolution spectrum of the Re@COP2 sample. Figure S5: O 1s high resolution spectrum of the Re@COP2 sample. Figure S6: N 1s high resolution spectrum of the Re@COP2 sample. Figure S7: Re 4f high resolution spectrum of the Re@COP2 sample. Figure S8: Cl 2p high resolution spectrum of the Re@COP1 sample. Figure S9: SEM images taken at 2 µm magnification of POP1, Re@POP1, POP2, and Re@POP2.
