# Supplementary material for: Microporous Polymelamine Framework Functionalized with Re(I) Tricarbonyl Complexes for CO2 Absorption and Reduction
Source: Polymers (Basel). 2022 Dec 14;14(24):5472. doi: 10.3390/polym14245472 (PMC9782493; doi:10.3390/polym14245472)
Supplement: Supplementary file 1 [file polymers-14-05472-s001.zip › polymers-2050914-supplementary.pdf]

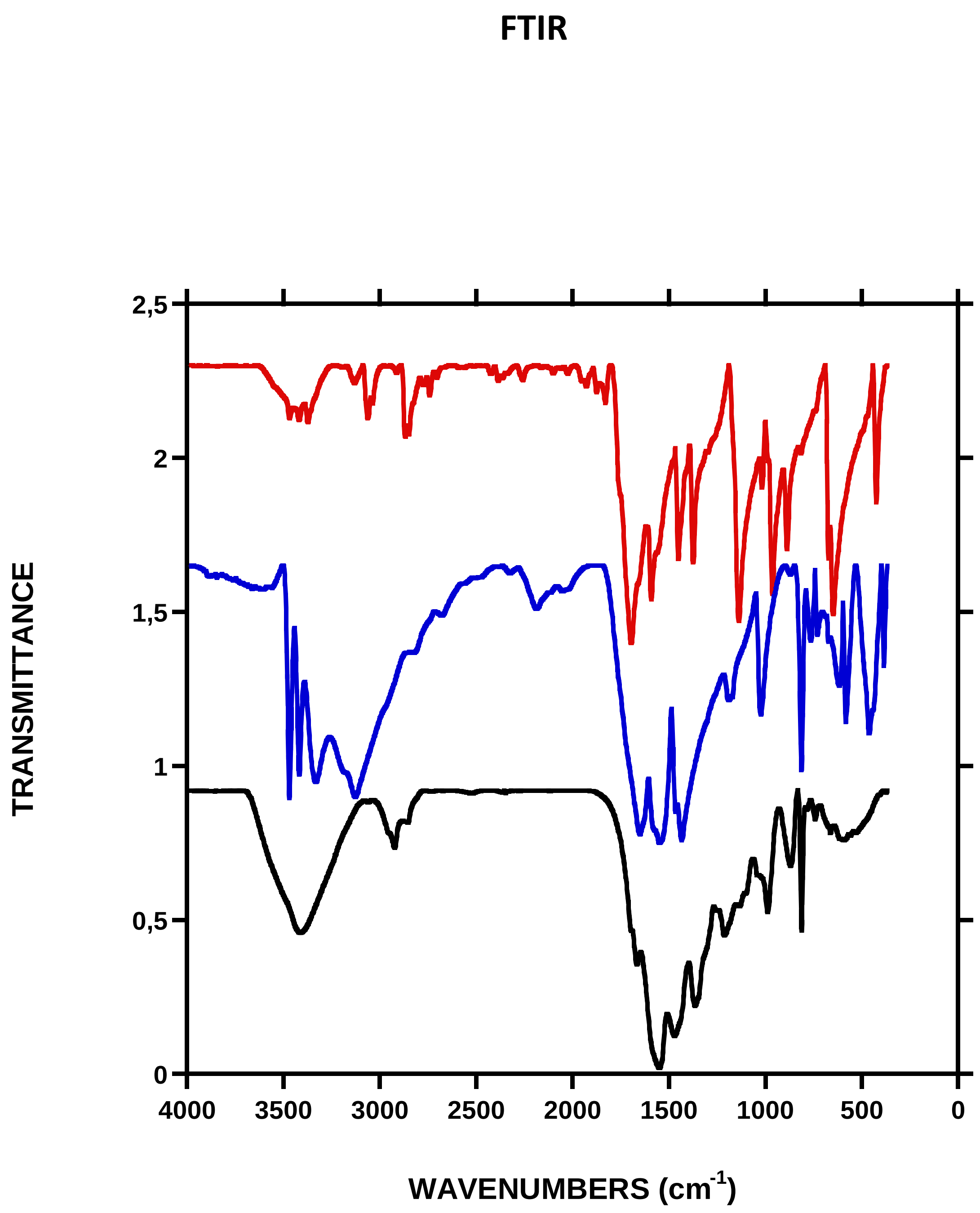

Figure S1. FTIR spectra of benzene-1,3,5-tricarboxaldehyde (red), melamine (blue), and POP (black). Applied shift in transmittance is due to ease the view of the spectra.

^{13}C CP/MAS NMR

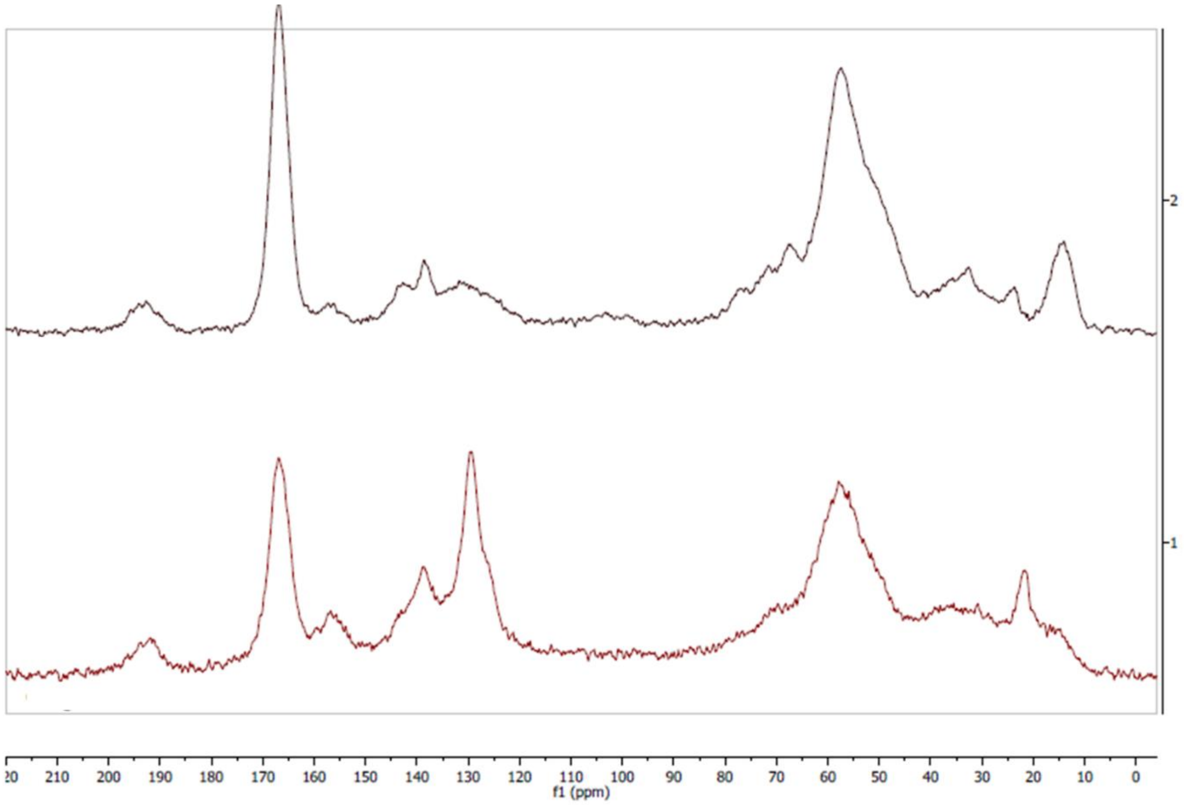

Figure S2. Solid state ^{13}C CP-MAS NMR spectrum for POP (above black trace) and Re@POP (below red trace)

ICP-OES

The stoichiometric composition of Re-polymer material is not defined but is known that Re content does not exceed 13% by weight. Consequently, taking the approximate model suggested in ref. 23 (see Scheme 1), an average molecular weight derived close to 934 is derived; by adding $Re(CO)_3Cl$ a total Mw over 1240 results. From this a Re content over 15% is calculated, to be compared with 22% derived from ICP-OES. It is concluded that *single* Rhenium per Re@POP molecule is present, clearly in the limit of the model approximation.

XPS of Re@POP2

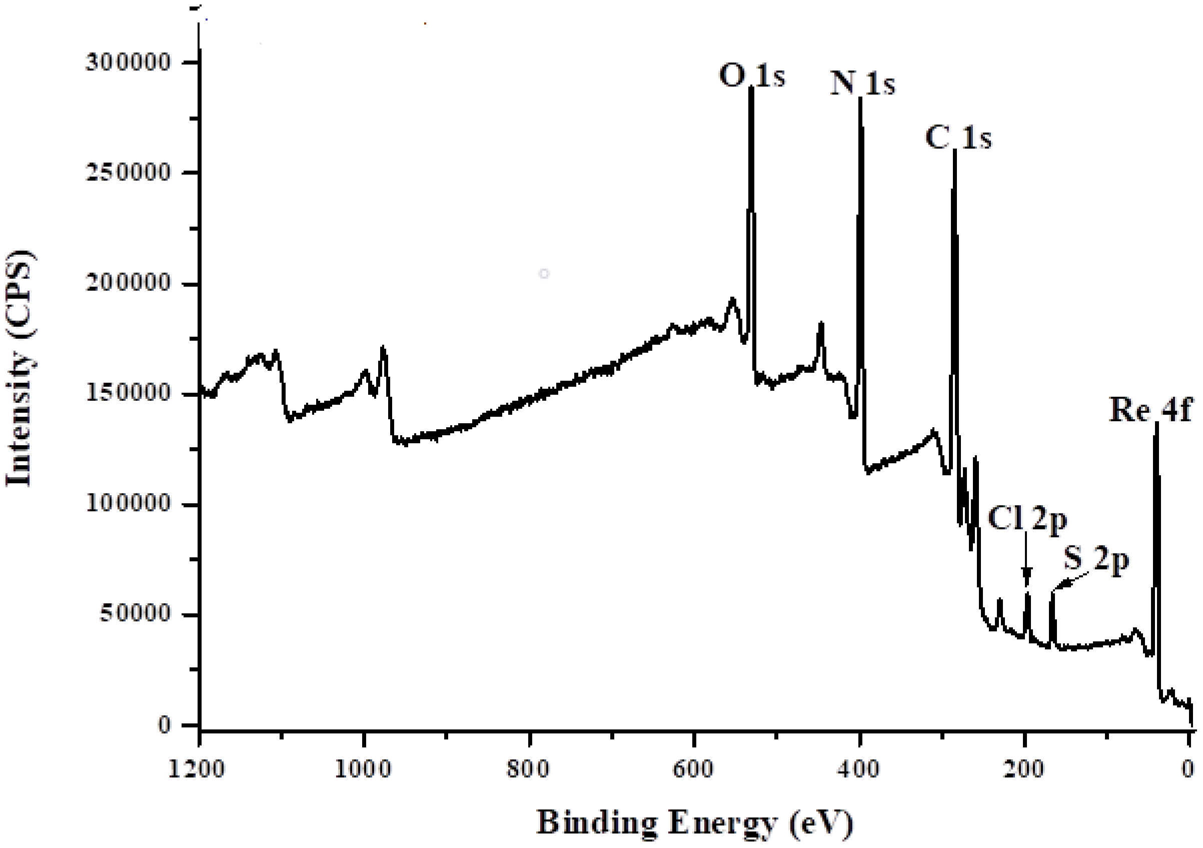

Figure S3. Wide scan XPS spectrum of the Re@POP2 sample

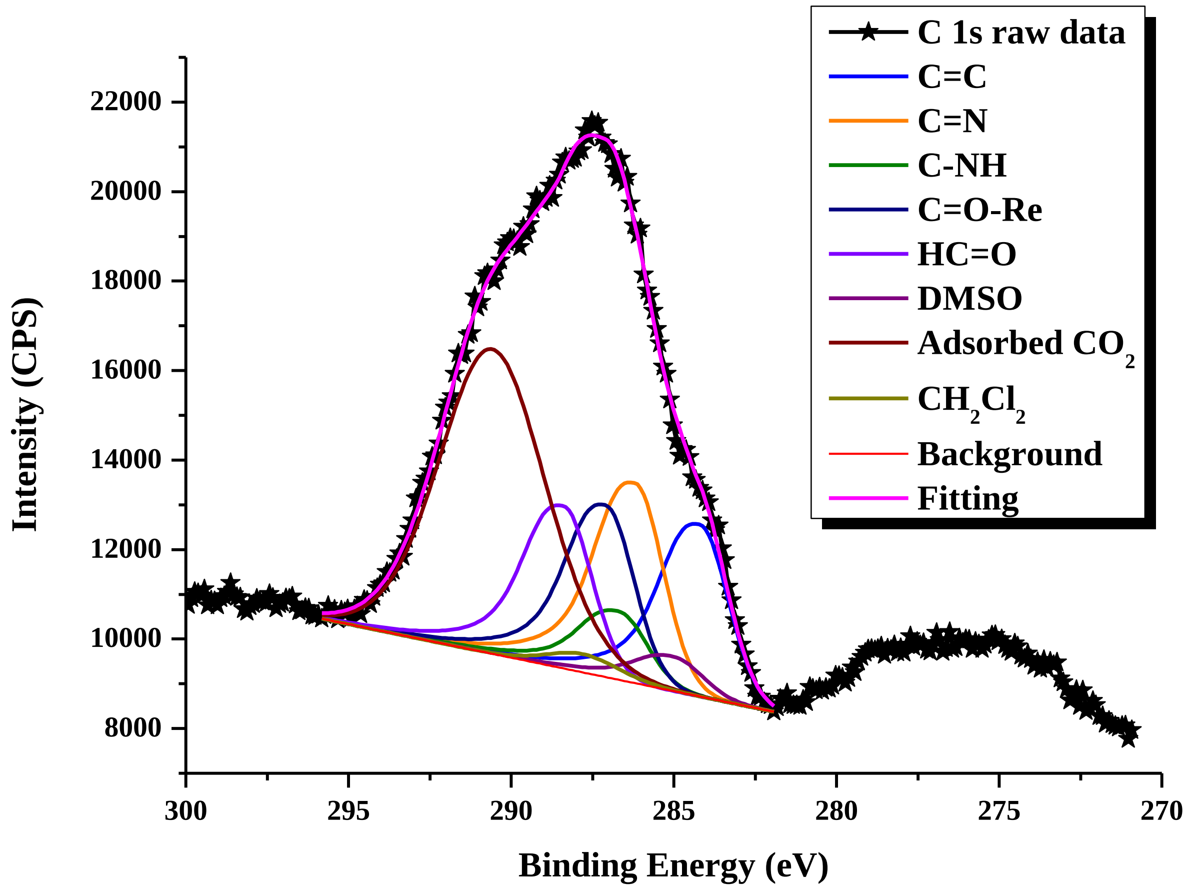

Figure S4. C 1s high resolution spectrum of the Re@POP2 sample

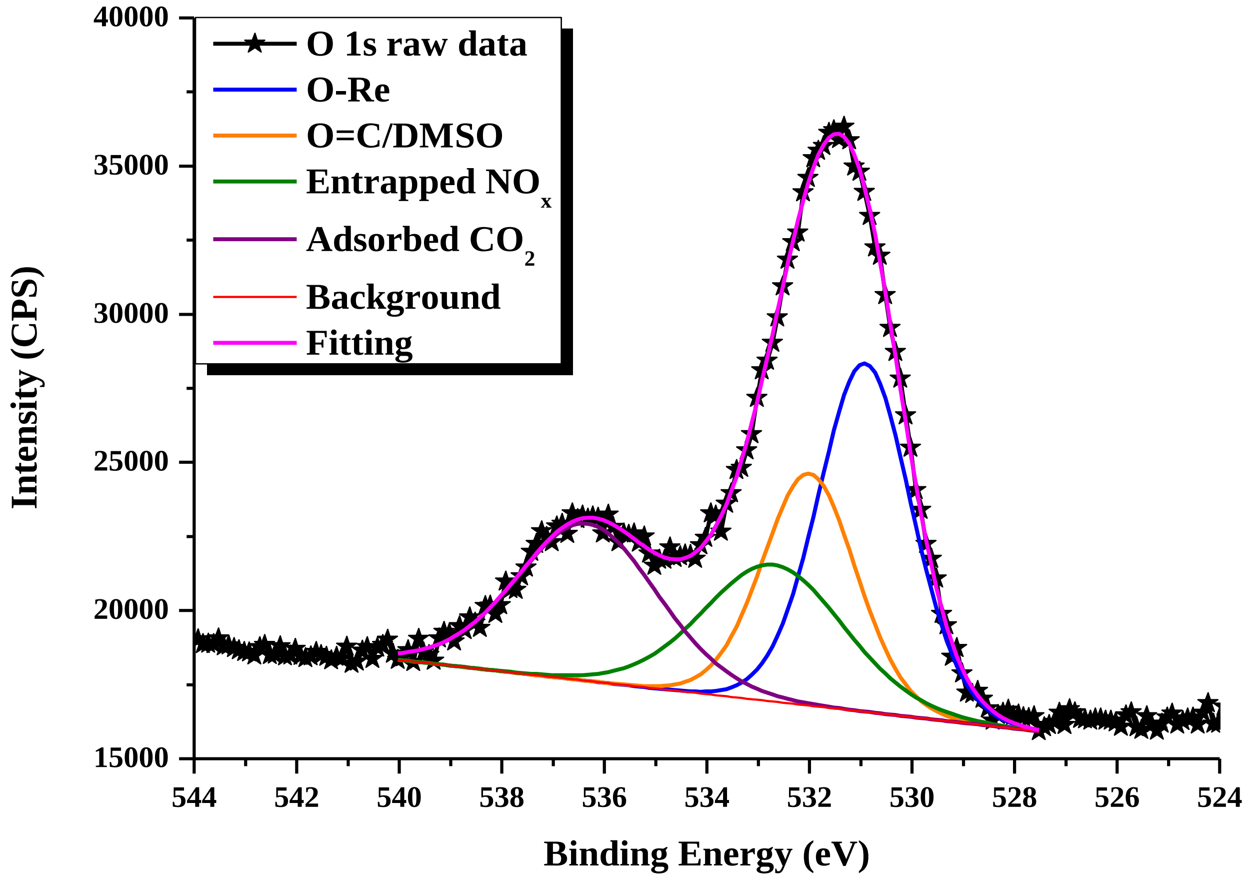

Figure S5. O 1s high resolution spectrum of the Re@POP2 sample

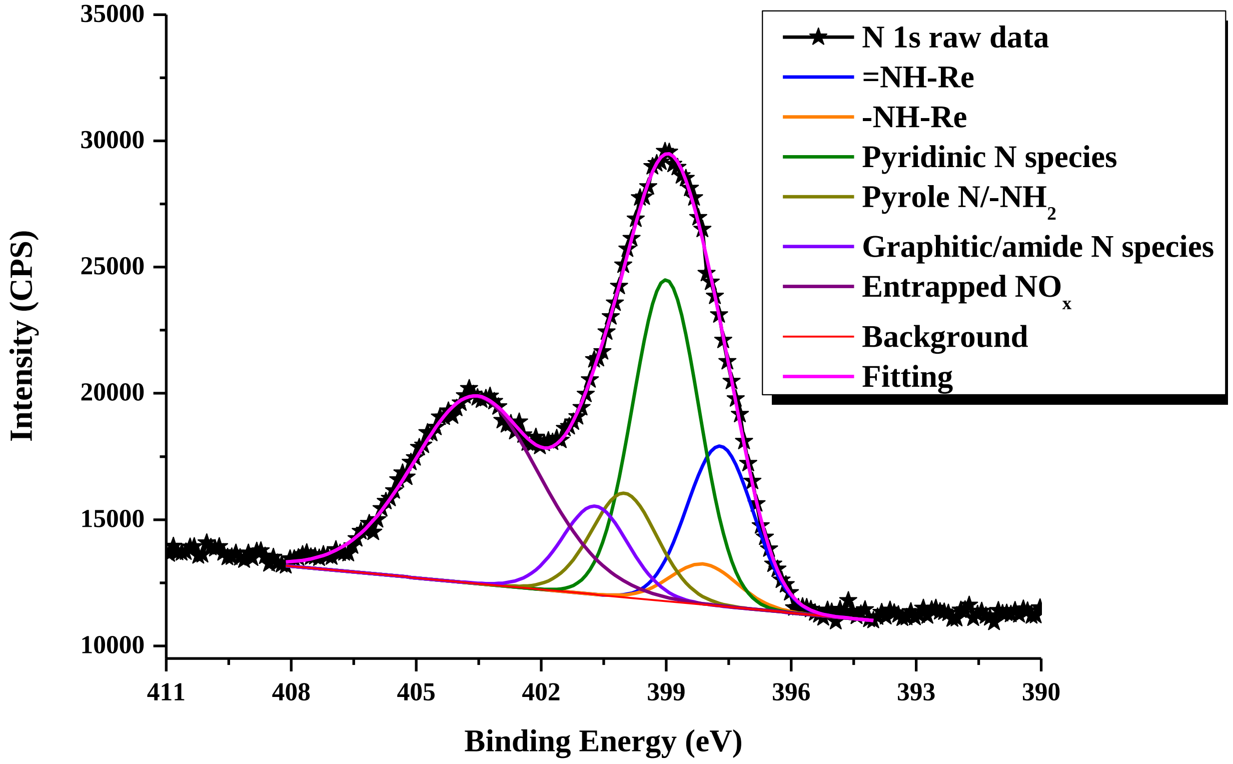

Figure S6. N 1s high resolution spectrum of the Re@POP2 sample

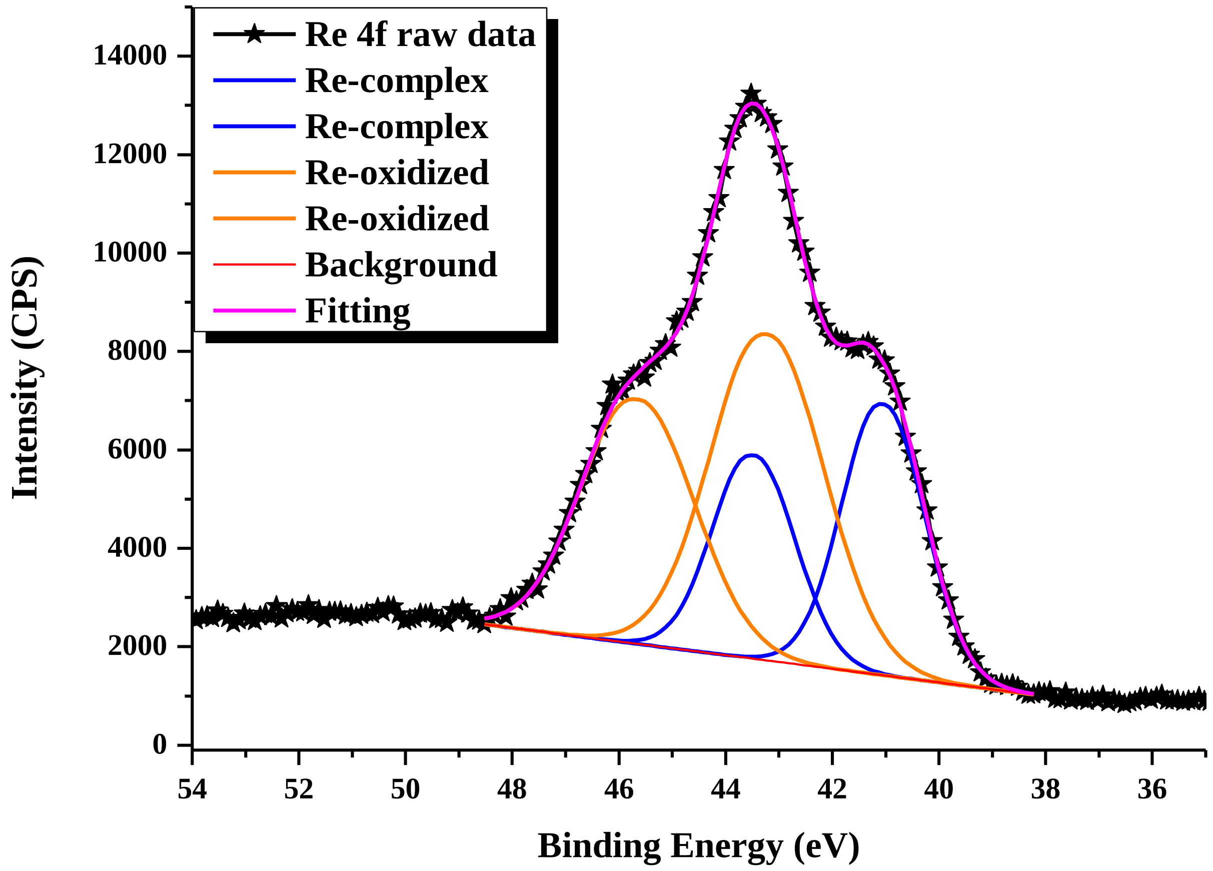

Figure S7. Re 4f high resolution spectrum of the Re@POP2 sample

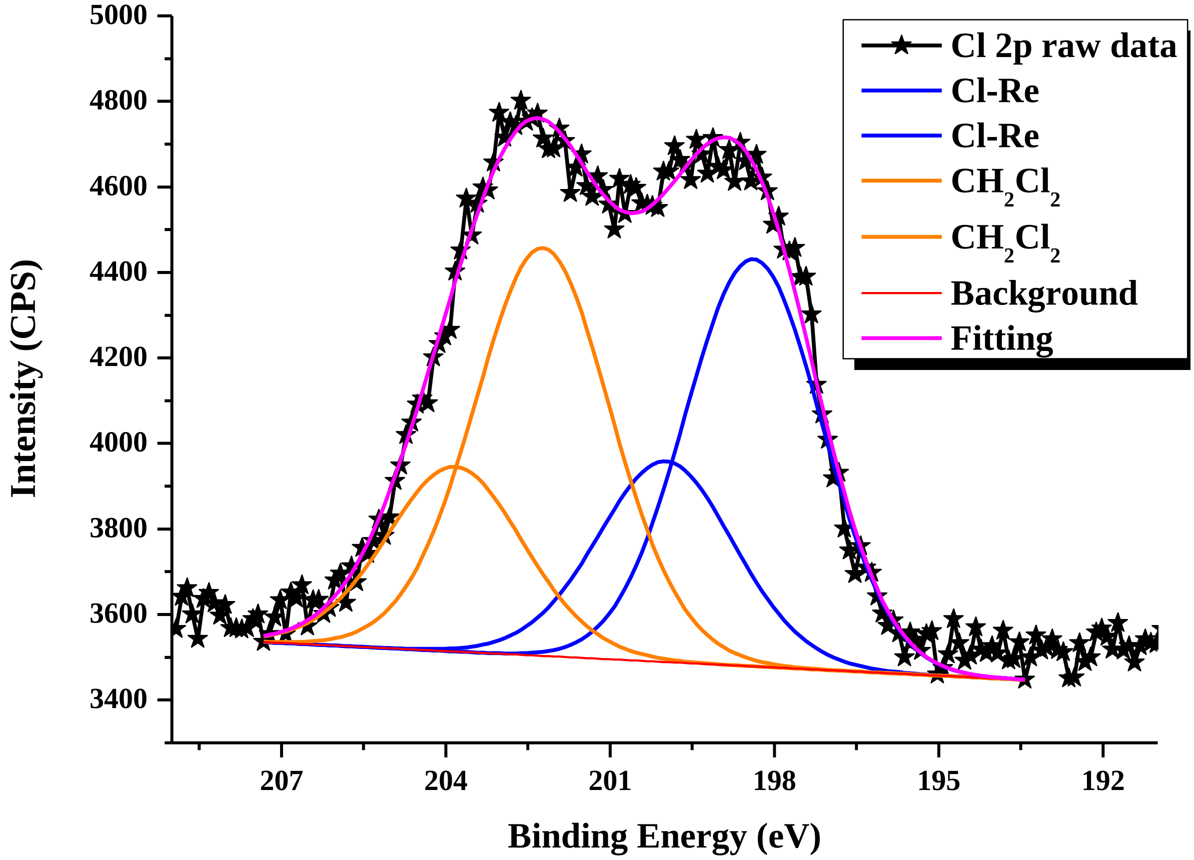

Figure S8. Cl 2p high resolution spectrum of the Re@POP1 sample

SEM IMAGES AT MEDIUM MAGNIFICATION

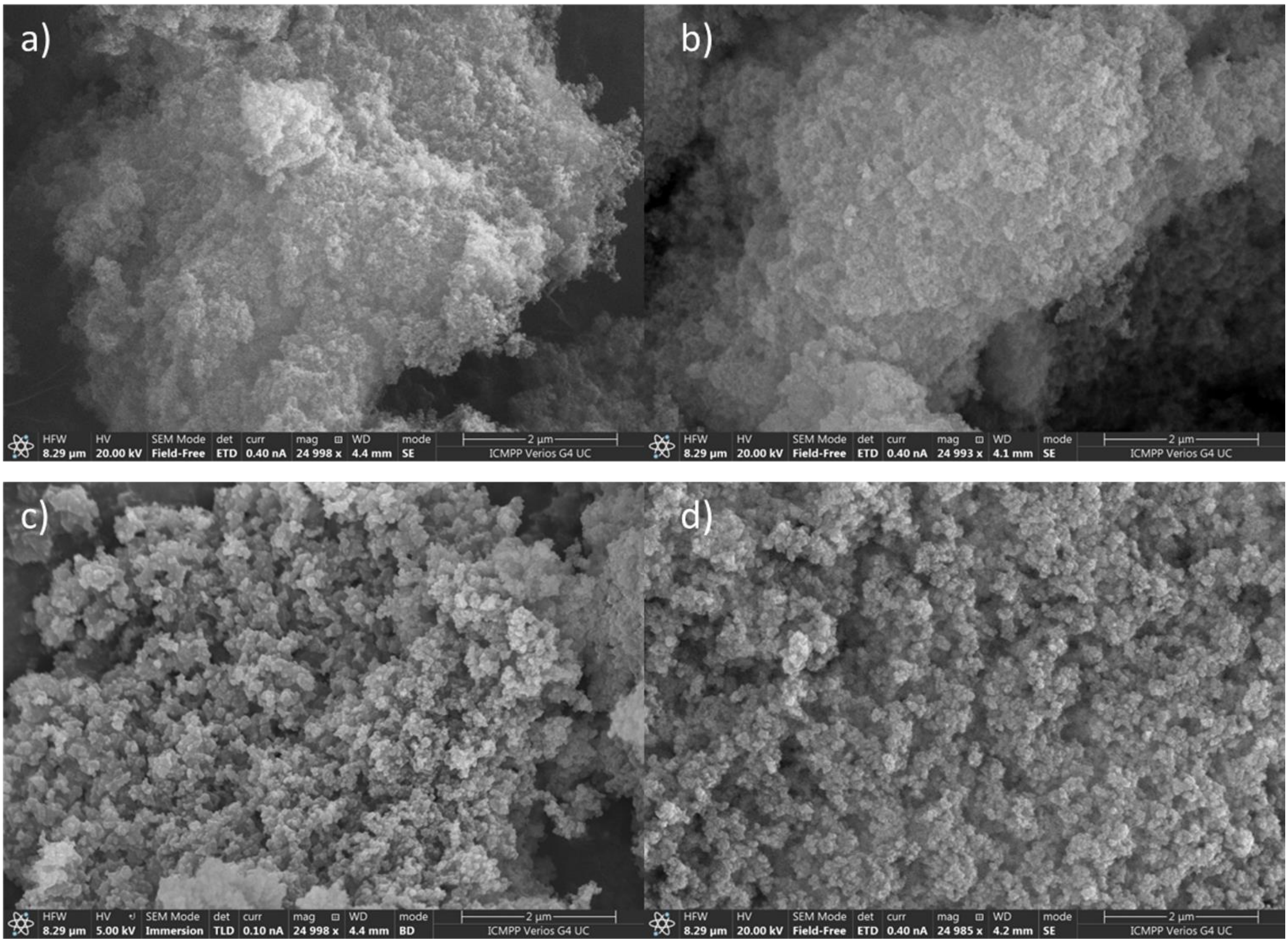

Figure S9. SEM images taken at 2 µm magnification. **a)** POP1, **b)** Re@POP1, **c)** POP2, **d)** Re@POP2.
